# Role of Transcription Factor Modifications in the Pathogenesis of Insulin Resistance

**DOI:** 10.1155/2012/716425

**Published:** 2011-10-26

**Authors:** Mi-Young Kim, Jin-Sik Bae, Tae-Hyun Kim, Joo-Man Park, Yong Ho Ahn

**Affiliations:** ^1^Department of Biochemistry and Molecular Biology, Yonsei University College of Medicine, 50 Yonsei-ro, Seodaemun-gu, Seoul 120-752, Republic of Korea; ^2^Center for Chronic Metabolic Disease Research, Yonsei University College of Medicine, 50 Yonsei-ro, Seodaemun-gu, Seoul 120-752, Republic of Korea; ^3^Brain Korea 21 Project for Medical Sciences, Yonsei University College of Medicine, 50 Yonsei-ro, Seodaemun-gu, Seoul 120-752, Republic of Korea

## Abstract

Non-alcoholic fatty liver disease (NAFLD) is characterized by fat accumulation in the liver not due to alcohol abuse. NAFLD is accompanied by variety of symptoms related to metabolic syndrome. Although the metabolic link between NAFLD and insulin resistance is not fully understood, it is clear that NAFLD is one of the main cause of insulin resistance. NAFLD is shown to affect the functions of other organs, including pancreas, adipose tissue, muscle and inflammatory systems. Currently efforts are being made to understand molecular mechanism of interrelationship between NAFLD and insulin resistance at the transcriptional level with specific focus on post-translational modification (PTM) of transcription factors. PTM of transcription factors plays a key role in controlling numerous biological events, including cellular energy metabolism, cell-cycle progression, and organ development. Cell type- and tissue-specific reversible modifications include lysine acetylation, methylation, ubiquitination, and SUMOylation. Moreover, phosphorylation and O-GlcNAcylation on serine and threonine residues have been shown to affect protein stability, subcellular distribution, DNA-binding affinity, and transcriptional activity. PTMs of transcription factors involved in insulin-sensitive tissues confer specific adaptive mechanisms in response to internal or external stimuli. Our understanding of the interplay between these modifications and their effects on transcriptional regulation is growing. Here, we summarize the diverse roles of PTMs in insulin-sensitive tissues and their involvement in the pathogenesis of insulin resistance.

## 1. Posttranslational Modifications of Transcription Factors: Relevance in the Context of Metabolic Syndrome

Transcription is the seminal event in the expression of genes and is a central point at which gene expression is regulated. Many cellular processes, including those that are tissue-specific or developmentally related, are largely controlled at the transcriptional level [1]. Transcription factors often regulate the expression of genes by binding to specific consensus sequences, or *cis* elements, within promoter regions [2]. Once bound, coregulators that either activate or repress transcription are recruited [3, 4]. Transcription factors play critical roles in regulating constitutive and inducible gene expression. In response to cellular stimuli, these proteins can be targets of modifications that affect their stability, activity, intracellular distribution, and interaction with other proteins [5]. Different external and internal signals direct distinct patterns of posttranslational modifications (PTMs), which transduce the signals for specific metabolic processes.

The number of people diagnosed with type 2 diabetes mellitus (T2DM) worldwide has been estimated to exceed 200 million [6]. Left untreated or uncontrolled, this disease can cause serious complications such as blindness, kidney damage, and vascular damage that may require the amputation of limbs or digits. T2DM is characterized by defects in both insulin sensitivity and secretion [7]. Central to this defect is insulin resistance, which reflects impaired sensitivity of target organs—primarily liver, pancreas, adipose tissue, and muscle—to insulin [8, 9]. Although the pathogenesis of insulin resistance remains unclear, abnormal insulin signaling [10], mitochondrial dysfunction [11], endoplasmic reticulum (ER) stress [12], dysfunctional triglyceride/free fatty acid cycle intermediates [13], and inflammation [14] have been reported to be involved in mediating this disease. These abnormalities lead to alterations in the transcription of key metabolic genes accompanied by PTMs of transcription factors that may result in the suppression or activation of target genes.

Recent advances in the understanding of PTMs, including those of transcription factors, have provided greater insight into the altered gene regulation that results in insulin resistance. Interestingly, multiple PTMs—both independent and interdependent—can occur, creating the potential for diverse cellular responses through changes at the transcriptional level. In this paper, we will limit our discussion to transcription factor PTMs responsible for metabolic alterations associated with insulin resistance.

## 2. Types of Transcription Factor Modifications

**Figure 1:**
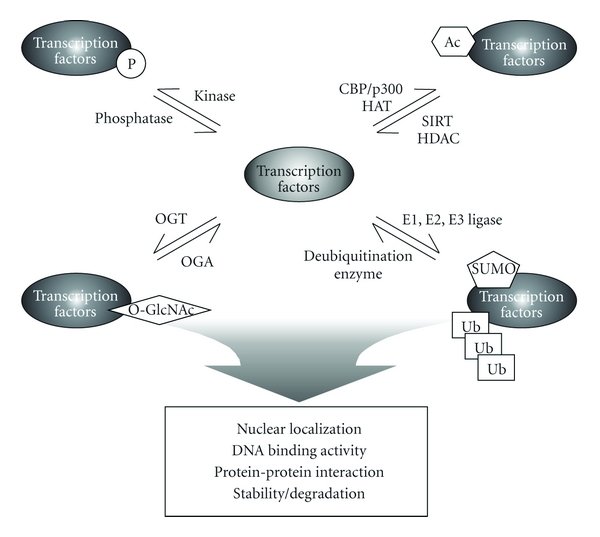
The types and functions of post-translational modification of transcription factors.

PTMs could be considered an evolutionary solution to the limited number of transcription factors, expanding the functional repertoire of genetic regulatory elements to cover the diverse metabolic requirements that are met through regulated gene expression. Although a large number of transcription factors have been demonstrated to be modified by PTM, there are still more left to be discovered. Furthermore, the interrelationship between various types of PTM should be understood in terms of modulating the DNA binding activity, stability, localization, and protein-protein interactions. Transcription factors can undergo several different types of PTMs, including acetylation, phosphorylation, glycosylation, and ubiquitination. The transcription factors and target genes considered in this paper are listed in Table 1. In addition, the functions of PTM of transcription factors are summarized in Figure 1.

### 2.1. Acetylation/Deacetylation

Acetylation of histone or nonhistone proteins is critical for gene expression. This modification, which occurs on lysine residues, affects protein stability, localization, degradation, and function. Moreover, this modification can also influence protein-protein and protein-DNA interactions. Interestingly, most acetylated forms of nonhistone proteins have been shown to be involved in tumorigenesis and immune function. Our understanding of the role of acetylation of transcription factors involved in insulin resistance is incomplete, but emerging evidence indicates that acetylation influences the subcellular distribution, DNA binding ability, and proteasomal degradation of these proteins [15].

### 2.2. Phosphorylation/Dephosphorylation

External stimuli often lead to the activation of signal transduction pathways that result in the phosphorylation of transcription factors. Depending on the stimulus, specific amino acid residues, typically tyrosine, serine, and/or threonine, are phosphorylated by one or more protein kinases. Dephosphorylation by phosphatases can also occur in response to cellular signals. This phosphorylation/dephosphorylation dynamic can directly regulate distinct aspects of transcription factor function, including subcellular distribution, DNA binding, transacting ability, and protein stability [16, 17]. 

### 2.3. Modification by O-Linked-N-Acetylglucosamine: O-GlcNAcylation

*O*-GlcNAcylation is a dynamic, inducible, and reversible, nutrient-sensitive post-translational event in which *O*-linked-*N*-acetylglucosamine (*O*-GlcNAc) is attached to serine and/or threonine hydroxyl groups of cytosolic [18], mitochondrial [19], or nuclear proteins [18] by the concerted actions of *O*-GlcNAc transferase (OGT) and *O*-GlcNAcase [18, 20]. 

UDP-GlcNAc is a major end product of the hexosamine biosynthesis pathway and functions as a cellular nutrient sensor. Sustained exposure to high concentrations of glucose and glucosamine increases UDP-GlcNAc levels, which, in turn, results in an increase in *O*-GlcNAc-glycosylated proteins and leads to glucotoxicity in various insulin-sensitive tissues [21]. Indeed, insulin-signaling molecules, including the *β* subunit of the insulin receptor, insulin receptor substrate (IRS)-1 and -2, the p85 and p110 subunits of phosphoinositide 3-phosphate kinase (PI3K), protein kinase B (PKB)/Akt, and 3-phosphoinositide-dependent protein kinase-1 (PDK1), are targets of OGT, and O-GlcNAcylation of these proteins causes downregulation of insulin signaling [22].

### 2.4. Ubiquitination and SUMOylation

The amount of intracellular protein is regulated by the rates of protein synthesis and degradation. In general, protein degradation occurs via the ubiquitin-proteasome pathway [23]. Ubiquitin, a highly conserved protein consisting of 76 amino acids, is targeted to substrate proteins and polymerized by the sequential action of three enzymes: E1, a ubiquitin-activating enzyme; E2, a ubiquitin-conjugating enzyme; E3, a ubiquitin-protein ligase [24]. The resulting protein contains multiple chains of branched ubiquitin molecules that enable recognition by the 26S proteasome, which subsequently mediates degradation of the ubiquitinated protein into small peptides [24, 25].

In addition to ubiquitination, transcription factors can also be modified by the addition of SUMO (small ubiquitin-related modifier), a protein composed of 97 amino acids. In this event, SUMO is attached to lysine residues in the substrate protein by the sequential action of three enzymes [26]. SUMOylation can affect protein stability, subcellular localization, or protein-protein interactions [27, 28]. SUMOylation often competes with ubiquitination and/or acetylation for lysine residues on target transcription factors [29, 30].

Reports have suggested that deregulated ubiquitin/proteasome-mediated degradation of insulin signaling molecules results in insulin resistance and the development of diabetes [31].

## 3. Modification of Transcription Factors in the Insulin-Sensitive Tissues

### 3.1. Liver Metabolism

#### 3.1.1. Effect of Transcription Factor Modifications on Hepatic Gluconeogenesis

**Figure 2:**
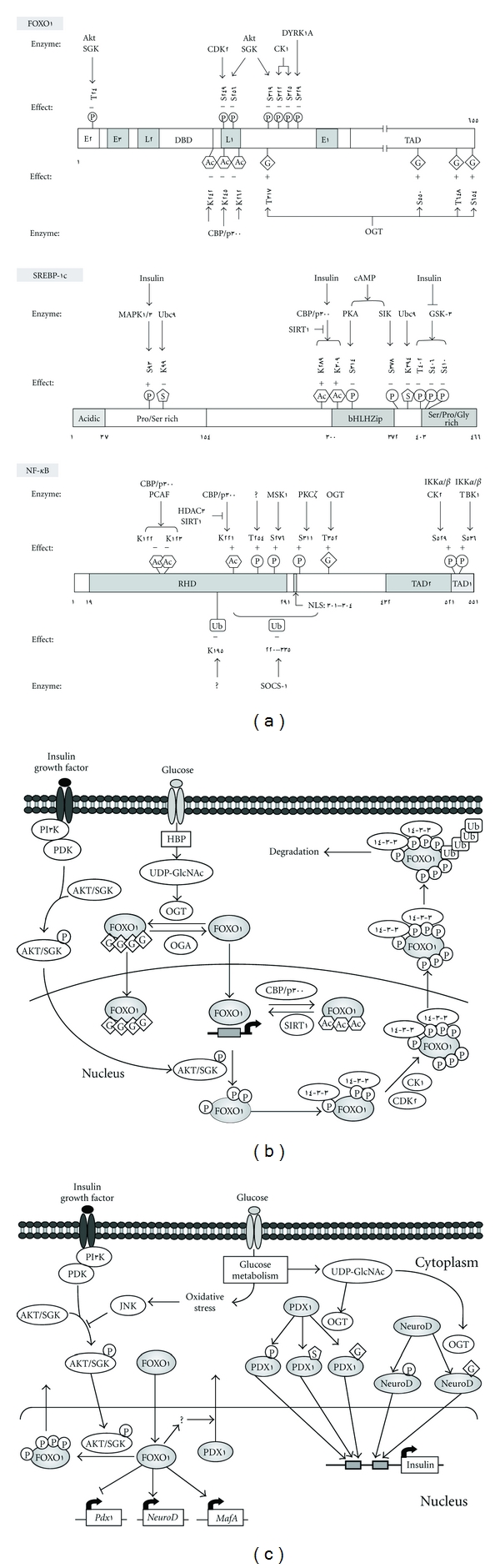
Post-translational modifications (PTMs) of transcription factors. (a) The positions of PTM sites in the human FOXO1, SREBP-1c, and NF-*κ*B p65 subunit.The positions of PTM sites and the implicated modifying enzymes are shown. (+) and (–) represent activation and inhibition of the transcriptional activity of transcription factors, respectively. L1-2, nuclear localization sequences; E1-3, nuclear export sequences; DBD, DNA-binding domain; TAD, transactivation domain; RHD, Rel homology domain; NLS, nuclear localization sequence; TAD, transactivation domain. (b) Regulation of FOXO1 nucleocytoplasmic shuttling and transcriptional activity by PTMs in liver. (c) Regulation of transcription factor activities by PTMs in pancreatic *β* cells. P, phosphate group; Ac, acetyl group; G, *O*-linked-*N*-acetylglucosamine; Ub, ubiquitin; S, SUMO; Akt, v-akt murine thymoma viral oncogene homolog 1 (also known as protein kinase B [PKB]); SGK, serum/glucocorticoid-regulated kinase; CK1, casein kinase 1; DYRK1A, dual-specificity tyrosine-phosphorylated and regulated kinase1 A; CDK2, cyclin-dependent kinase 2. PI3K, phosphoinositide-3-kinase; PDK, phosphatidylinositol-dependent protein kinase; OGT, *O*-linked N-acetylglucosamine (GlcNAc) transferase; MAPK1/3, mitogen-activated protein kinase 1/3; Ubc9, ubiquitin conjugating enzyme 9; p300, E1A-binding protein p300; CBP, CREB-binding protein; SIRT1, sirtuin 1; PKA, protein kinase A; cAMP, cyclic adenosine monophosphate; SIK, salt-inducible kinase; GSK-3, glycogen synthase kinase-3; JNK, c-Jun N-terminal kinase; PCAF, CBP/p300-associated factor; MSK1, mitogen/stress-activated protein kinase 1; PKC*ζ*, protein kinase C*ζ*; IKK, I kappa B kinase; CK2, casein kinase 2; TBK1, tank-binding kinase 1; SOCS-1, suppressor of cytokine signaling 1; HBP, hexosamine biosynthesis pathway; OGA, *O*-GlcNAcase; PDX1, pancreatic and duodenal homeobox 1; NeuroD, neurogenic differentiation; MafA, v-maf (maf musculoaponeurotic fibrosarcoma) oncogene homolog A.

Hepatic gluconeogenesis is an essential process during fasting or starvation. However, activation of gluconeogenesis in patients with T2DM causes hyperglycemia. Insulin has been shown to suppress gluconeogenesis in the liver [32]. When insulin binds to its receptor, signal transduction pathways are activated that lead to the induction of Akt, which phosphorylates the Forkhead protein, FOXO1 [33, 34], a major transcription factor for gluconeogenic gene expression. The phosphorylated form of FOXO1 is translocated from the nucleus to the cytosol (Figure 2(b)).

FOXO proteins have been reported to modulate a variety of cellular responses depending on the cell type [35]. Subfamilies of FOXO proteins include FOXO1 (FKHR), FOXO3a (FKHR-like1), and FOXO4/AFX (acute lymphocytic leukemia-1 fused gene from chromosome X). FOXO1 is a positive *trans* acting factor that binds to promoter regions within the glucose-6-phosphatase (*G6pc*) [36], phosphoenolpyruvate carboxykinase (*Pck1*) [37], and peroxisome proliferator-activated receptor-coactivator-1 alpha (*Ppargc1a*) genes [38]. Composed of 655 amino acids, FOXO1 contains seven phosphorylation sites, namely Thr^24^, Ser^249^, Ser^256^, Ser^319^, Ser^322^, Ser^325^, and Ser^329^, which are modified by a variety of mechanisms (Figure 2(a)). Thr^24^, Ser^256^, and Ser^319^ are phosphorylated by protein kinase B (PKB)/Akt (v-akt murine thymoma viral oncogene homolog 1) in response to insulin/insulin growth factor-1 signaling [39]. Ser^249^ is phosphorylated by CDK2 (cyclin-dependent kinase 2) [40], whereas Ser^322^ and Ser^325^ are phosphorylated by CK1 (casein kinase 1) [41]. Lastly, Ser^329^ is phosphorylated by the dual-specificity kinase, DYRK1A (dual-specificity tyrosine-phosphorylated and regulated kinase 1A) [42]. 

As a result of Thr^24^, Ser^256^, and Ser^319^ phosphorylation [39], FOXO1 is exported from the nucleus to the cytoplasm [43] where it binds 14-3-3 proteins. Once bound, FOXO1 is retained in the cytoplasm and targeted for proteasomal degradation, preventing its reentry into the nucleus (Figure 2(b)) [44–46]. Thus, phosphorylation and ubiquitination are important post-translational modifications of FOXO1 that are critical for its degradation and, ultimately, its regulation.

The transcriptional activities of FOXO1 are also controlled by its acetylation status. Acetylation by cAMP-response element-binding protein-binding protein (CBP) attenuates FOXO1 transcriptional activity [47]. Several acetylation sites have been identified in FOXO1, namely, Lys^242^, Lys^245^, and Lys^262^ [48] (Figure 2(a)). Following acetylation, the positive charges associated with these lysine residues are eliminated, inhibiting FOXO1 interaction with DNA and reducing the ability of this transcription factor to recognize its own *cis* element, including the insulin-response element, in some target genes [15]. In addi**t**ion, FOXO1 acetylation has been linked with increased phosphorylation at Ser^253^ by Akt [48, 49], which further decreases DNA binding. This indicates that the interplay between two types of PTMs regulates the DNA binding activity of FOXO1. On the contrary, deacetylation of FOXO1 is catalyzed by Sirtuin 1 (SIRT1), an NAD(+)-dependent deacetylase [47]. The transcriptional activity of FOXO1 is enhanced by resveratrol-activated SIRT1 resulting in the increase in the hepatic gluconeogenesis [50, 51]. 

A positive correlation between *O*-GlcNAcylation and insulin resistance has been demonstrated. Because *O*-GlcNAc modifications can also occur on many phosphorylation sites, it has been postulated that increased *O*-GlcNAc may hinder phosphorylation events that normally occur as a result of insulin signaling. This altered regulation can lead to insulin resistance [52]. Indeed, serine and threonine residues within FOXO1 have been shown to be modified by *O*-GlcNAcylation (Figure 2(a)), resulting in increased transcription of *G6pc *and *Ppargc1a*, as well as genes involved in the detoxification of reactive oxygen species (ROS) [53–55]. This effect is independent of FOXO1 subcellular distribution [53]. Presumably, FOXO1 glycosylation could cause a conformational change in FOXO1 and affect its affinity for DNA, which would have an impact on its intrinsic activity and interaction with other cofactors [54]. Modification of FOXO1 by *O*-GlcNAcylation has been observed in the liver of streptozotocin-induced diabetic animals, suggesting that this modification may be associated with hyperglycemia [53]. Indeed, chronic hyperglycemia can lead to hyperglycosylation of FOXO1, thus inducing *G6pc* [53], *Pck1* [54] and *Ppargc1a* genes [55], and causing further production of hepatic glucose. These observations suggest that FOXO1 *O*-GlcNAcylation is a major underlying cause of hepatic glucose overproduction in T2DM [53]. In the hyperglycemic state, *O*-GlcNAcylated PGC-1*α* recruits OGT to FOXO1; the associated OGT glycosylates FOXO1 and increases its transcriptional activity [56]. 

cAMP-response-element- (CRE-) binding protein (CREB) is another important transcription factor that stimulates gluconeogenesis. CREB directly binds to the promoters of *G6pc* and *Pck1* genes or increases gluconeogenesis by upregulating *Ppargc1a* gene expression [57]. CREB is phosphorylated at Ser^133^ in the transactivation domain by cAMP-dependent protein kinase (PKA), a modification that increases CREB transcriptional activity [58, 59]. As its name suggests, CREB is phosphorylated and activated in response to hormonal stimuli (e.g., glucagon) that activate adenylyl cyclase and thereby increase the intracellular concentration of cAMP. Binding of cAMP to PKA releases the catalytic domain of PKA from the holoenzyme, allowing it to translocate to nucleus and phosphorylate CREB [60]. In addition, phosphorylation of CREB at Ser^133^ promotes association with CBP/p300 [61] which upregulates CREB target gene expression by acetylating nucleosomal histones [62, 63] and recruiting RNA polymerase II complexes [64, 65]. By contrast, CaMKII (calcium- and calmodulin-dependent kinase II) induces phosphorylation at Ser^142^ in the transactivation domain [66], a modification that inhibits CREB activity by disrupting CREB interaction with CBP/p300 [67]. DNA damage-mediated phosphorylation of CREB at Ser^111^ and Ser^121^ by AMT (ataxia-telangiectasia mutated) also inhibits CREB activity by blocking CREB-CBP interaction [68, 69]. 

CRTC2 (CREB-regulated transcription coactivator 2) interacts with the bZIP domain of CREB and thereby induces its activity [70, 71]. The resulting CRTC2-CREB complex binds to *cis* elements in the promoters of *G6pc, Pck1*, and *Ppargc1a* genes [72, 73]. CRTC2 is also regulated by O-GlcNAcylation [74]. Further research is needed to elucidate the molecular mechanisms and site-specific roles of O-GlcNAcylation in relation to phosphorylation or other types of PTMs in terms of glucotoxicity, insulin resistance, and T2DM.

#### 3.1.2. Modification of Transcription Factors That Regulate Lipid Metabolism Genes

NAFLD has become a common chronic disease due to western style diets. This disease manifests as a simple accumulation of triglycerides in hepatocytes (hepatic steatosis) or as steatohepatitis, which is accompanied by inflammation, fibrosis, cirrhosis, and hepatocellular carcinoma in severe cases. It has now become clear that accumulation of triglycerides in hepatocytes is correlated with T2DM, obesity, and insulin resistance. Steatosis is caused by an imbalance between lipid availability and disposal. Triglyceride accumulation in hepatocytes reflects dietary fatty acid intake, increased lipolysis in adipose tissue, or de novo lipogenesis. On the other hand, hepatic triglyceride levels are decreased by *β*-oxidation of fatty acid in the hepatocytes and triglyceride secretion with very low-density lipoproteins (VLDLs). In nonalcoholic fatty liver disease patients, the ratio of lipogenesis to VLDL-packaged triglyceride secretion is up to 25–30%, a substantial increase compared to the normal range of 2–5% [75, 76]. 

The expression of lipogenic enzymes is mainly controlled at the transcriptional level in the hyperinsulinemic and hyperglycemic state. Two major transcription factors, sterol regulatory element binding protein-1c (SREBP-1c) and carbohydrate response element binding protein (ChREBP), are well known to be involved in these states [77]. 

SREBP-1c is a member of the basic-helix-loop-helix-leucine zipper (bHLH-LZ) family of transcription factors. It is synthesized as an inactive form embedded in the membranes of the ER and is activated in the Golgi apparatus by proteolytic cleavage. The resulting N-terminal domain cleavage fragment (nSREBP-1c), which is the transcriptionally active form, is translocated to the nucleus. SREBP1a, which is expressed from an mRNA that overlaps that of SREBP-1c and differs from SREBP-1c only at the N-terminus, and SREBP-2, which is the product of a separate gene, regulate the expression of cholesterol synthesis genes [78]. Expression of the SREBP-1c gene and maturation and stability of SREBP-1c protein are regulated by insulin through the PI3K-PDK1-PKB/Akt pathway [79, 80]. PKB/Akt kinase phosphorylates and inhibits glycogen synthase kinase-3 (GSK3), whereas the dephosphorylated form of GSK3 phosphorylates Thr^426^, Ser^430^, and Ser^434^ of nSREBP-1a, causing degradation by ubiquitination through the ubiquitin ligase, FBW7 (F-box and WD repeat domain containing 7) [81]. Similarly, phosphorylation of nSREBP1c has been reported [81, 82]. Ser^117^ of SREBP-1a and Ser^93^ of SREBP-1c are phosphorylated by mitogen-activated protein kinase 1/3, and mutation of these sites abolishes insulin-induced transcriptional activity (Figure 2(a)) [83].

By contrast, cAMP might act through PKA to regulate SREBP-1c processing. Phosphorylation of Ser^338^ of SREBP-1a and Ser^314^ of SREBP-1c by PKA reduces the transcriptional activities of the corresponding transcription factors (Figure 2(a)) [84]. In addition, the nonhydrolyzable PKA activator, dibutyryl-cAMP, downregulates the proteolytic processing of SREBP-1a [85]. These results indicate that insulin and glucagon also modulate the transcriptional activity of SREBP-1c through phosphorylation. Salt-inducible kinase, a member of the AMP-activated protein kinase (AMPK) family, phosphorylates Ser^329^ of SREBP-1a and reduces lipogenic gene expression (Figure 2(a)) [86].

Modification of SREBP-1a at Lys^123^ and Lys^418^ by Ubc9, an SUMO-1-conjugating enzyme, reduces its transcriptional activity (Figure 2(a)). However, ubiquitination and SUMOylation do not compete for the same Lys residues, and SUMOylation does not affect ubiquitination-mediated SREBP degradation and stability [87].

CBP/p300-mediated acetylation of SREBP-1c increases its stability [88]. Lys^289^ and Lys^309^ residues near and within the DNA-binding domain of SREBP-1c, respectively, are acetylated by p300 and deacetylated by SIRT1 (Figure 2(a)) [89]. Levels of acetylated SREBP-1c are increased in fed mice, diet-induced obese mice, and insulin- and glucose-treated HepG2 cells. SIRT1 overexpression decreases SREBP-1c acetylation level and protein stability, causing a reduction in lipogenic gene expression [89].

ChREBP, which is also a member of the bHLH-LZ (leucine zipper) family of transcription factors, is the second of the two major transcription factors shown to induce glycolytic and lipogenic genes in hepatocytes [90]. ChREBP, also known as MLXIPL (MLX interacting proteinlike), forms a heterodimer with the bHLH-LZ protein Mlx (MAX-like protein X) that binds the carbohydrate response element of various glucose-responsive genes, including liver type pyruvate kinase (*Pklr*), fatty acid synthase (*Fasn*), and acetyl-CoA carboxylase 1 (*Acc1*) [91]. Nuclear localization of ChREBP is induced by high glucose. In starvation, glucagon increases intracellular cAMP concentrations and activates PKA. Phosphorylation of ChREBP by PKA at Ser^196^ prevents nuclear localization, whereas PKA-mediated phosphorylation at Thr^666^ inhibits DNA binding [92]. In addition, phosphorylation of Ser^568^ of ChREBP by AMPK decreases ChREBP transcriptional activity [93]. In contrast, xylulose-5-phosphate generated from glucose through the hexose monophosphate shunt activates protein phosphatase 2A delta, which dephosphorylates ChREBP and increases lipogenesis [94]. However, the regulation of ChREBP by phosphorylation and dephosphorylation remains controversial [95, 96]. 

A recent study has shown that by increasing the stability and transcriptional activity of ChREBP, O-GlcNAcylation of ChREBP in the hyperglycemic state is responsible for fatty acid synthesis in the mouse liver [97].

### 3.2. *β*-Cell Dysfunction and Pancreatic Failure

The pancreas maintains normal blood glucose levels by regulating insulin and glucagon secretion. Insulin, an anabolic hormone, modulates a variety of biological processes and metabolic pathways, including cell survival and proliferation, glycogen synthesis, protein synthesis, and glucose uptake into skeletal muscle and adipocytes. In an attempt to overcome the reduction in insulin activity that occurs during insulin resistance, the number of *β* cells increases, resulting in a compensatory hypersecretion of insulin. As the compensation fails, the *β*-cell phenotype is disturbed, causing a reduction in *β*-cell mass via apoptosis [98].

FOXO1 has been shown to modulate pancreatic *β*-cell development, proliferation, maintenance, expansion, and apoptosis [99, 100]. *β*-cell failure was observed in IRS2-deficient mice [101] and FOXO1^S253A^ transgenic mice [102] which exhibited decreased or nonfunctional FOXO1 phosphorylation, respectively. Interestingly, FOXO1 haplodeficiency partially restored *β*-cell proliferation in these mice and increased the expression of pancreatic and duodenal homeobox 1 (*Pdx1*) [101] (Figure 2(c)), a critical transcription factor involved in *β*-cell differentiation, development, and cellular function [103]. In addition, by binding the Foxa2 site within the *Pdx1* promoter, FOXO1 can inhibit the expression of this crucial transcription factor [101].

FOXO1 also regulates the subcellular distribution of PDX1 [104] (Figure 2(c)). Nucleocytoplasmic translocation of PDX1 during hyperglycemia-induced oxidative stress occurs in a Jun N-terminal-kinase- (JNK-) dependent manner, resulting in *β*-cell failure [105]. JNK activation during these conditions results in decreased Akt activity and subsequent FOXO1 hypophosphorylation, leading to PDX1 translocation to the cytosol [104]. In support of this, infection of HIT-T15 cells with adenovirus expressing wild-type FOXO1 led to PDX1 translocation from the nucleus to the cytosol in the absence of H_2_O_2_ treatment [104]. The mechanism by which nuclear FOXO1 affects PDX1 translocation remains unknown although reports have suggested that the acetylation status of the two proteins may be responsible [104]. 

Acetylation and deacetylation of FOXO1 are modulated by CBP/p300 and SIRT1, respectively. Transgenic mice bearing a pancreatic *β*-cell-specific, SIRT1-overexpressing transgene (BESTO) display improved glucose tolerance and enhanced glucose-stimulated insulin secretion [106]. In addition, oxidative stress-mediated FOXO1 deacetylation induces the expression of neurogenic differentiation (*NeuroD*) and v-maf (mafmusculoaponeurotic fibrosarcoma) oncogene homolog A (*MafA*) [107], which play roles in preserving insulin secretion in response to glucose and thereby promote *β*-cell compensation. However, the deacetylated form of FOXO1 is more easily degraded by ubiquitination than the acetylated form, suggesting that acetylation status regulates the stability and transcriptional activity of this protein. In contrast, deacetylation of the phosphorylation-defective ADA-FOXO1 mutant, which is constitutively nuclear by virtue of mutation of Thr^24^ and Ser^316^ to Ala(A) and Ser^253^ to Asp(D), does not affect transcriptional activity [107], indicating that the transcriptional activity of FOXO1 is independent of its phosphorylation status.

In the pancreas, glucose-induced insulin gene transcription is mediated by three *β*-cell-specific transcription factors: NeuroD1, PDX1, and MafA [103]. NeuroD1 and PDX1 are *O*GlcNAcylated and translocated to nucleus under high-glucose conditions, exhibiting increased DNA-binding activity and promoting insulin gene expression and insulin secretion in mouse insulinoma 6 (MIN6) cells [108, 109]. In addition, in the Gato-Kakizaki rat model of T2DM, the levels of *O*-GlcNAcylated proteins, especially those of PDX1 and *O*-GlcNAc transferase, were elevated in whole pancreas and islets of Langerhans [110]. 

The transcriptional activities of both PDX1 and NeuroD1 are regulated by phosphorylation upon glucose stimulation [111, 112]. In response to glucose and insulin stimulation, PDX1 is phosphorylated by stress-activated protein kinase 2 (SAPK2); phosphorylation by PI3K induces nuclear translocation and transcriptional activation [113–115]. SUMOylation causes nuclear translocation of PDX1 and increases its stability [116]. In contrast, phosphorylation of Ser^61^ and/or Ser^66^ by GSK3 during oxidative stress promotes PDX1 degradation [117].

### 3.3. Inflammatory Response of Macrophages

One of the risk factors for obesity-induced insulin resistance and diabetes is inflammation. Inflammatory gene expression in hepatocytes induces insulin resistance [118]. Hepatic steatosis often accompanies abdominal adiposity, and inflammation plays a pivotal role in the progression of nonalcoholic fatty liver disease. In the obese state, increased proinflammatory substances from abdominal fat might initiate hepatic inflammation and steatosis [119], highlighting the importance of understanding the role of macrophages in the initiation of obesity-induced insulin resistance in adipose tissue. Enlargement of adipose tissue as a result of excess dietary intake induces hypoxic conditions and ER stress, which are accompanied by nuclear factor-kappa B (NF-*κ*B)- and JNK1-mediated upregulation of inflammatory genes [120, 121]. 

Once activated, NF-*κ*B and JNK1 increase the production of various cytokines and chemokines from adipocytes, including tumor necrosis factor (TNF)-*α*, interleukin (IL)-6, monocyte chemotactic protein (MCP)-1, and plasminogen activator inhibitor-1. These molecules play key roles in the recruitment and infiltration of macrophages into adipocytes [122–125]. In fact, IL-6 has been reported to regulate the development of insulin resistance [126]. In addition, MCP-1 has been reported to increase during high-fat diet-induced obesity, thereby contributing to macrophage infiltration into adipose tissue [127]. Macrophages produce proinflammatory cytokines that amplify the inflammatory state in neighboring adipocytes, leading to the secretion of other mediators, such as adipokines and free fatty acids. Free fatty acids enter the circulation to promote insulin resistance in hepatocytes and myocytes [128, 129]. 

NF-*κ*B is a master regulator of the expression of genes involved in the inflammatory response. NF-*κ*B is a multisubunit protein variably consisting of p50, p52, p65, c-Rel, and Rel B; p65 is the major target of protein modification [130] (Figure 2(a)). This subunit is acetylated at Lys^221^ by CBP/p300 and deacetylated by histone deacetylase 3 or SIRT1 during inflammation [131, 132]. NF-*κ*B is also a key mediator of TNF-*α*-induced IL-6 gene expression [131, 133]. Notably, an SIRT1 activator was shown to attenuate the TNF-*α*-induced inflammatory signal. Conversely, SIRT1 knockdown in 3T3-L1 adipocytes using small inhibitory RNAs increased NF-*κ*B acetylation and enhanced the transcription of inflammatory genes, causing insulin resistance [134, 135]. By contrast, acetylation of Lys^122^/Lys^123^ of the p65 subunit by CBP/p300 or CBP/p300-associated factor (PCAF) decreased NF-*κ*B DNA-binding ability and promoted NF-*κ*B nuclear export and interaction with I*κ*B*α*, ultimately, attenuating its transcriptional activity [136, 137]. Taken together, these results indicate that acetylation of specific lysine residues on p65 confers different functional consequences. 

Another modification that occurs on p65 is phosphorylation. Mitogen- and stress-activated protein kinase-1 (MSK1) is a nuclear kinase that phosphorylates Ser^276^ of p65. Treatment of cells with the MSK1 inhibitor H89 has been shown to block TNF-*α*-induced phosphorylation of p65 *in vivo*. TNF-*α* promotes the interaction between p65 and MSK1, which is recruited to the IL-6 promoter [138]. P65 can also be phosphorylated by protein kinase C*ζ* (PKC*ζ*) through TNF-*α* signaling. Phosphorylation of p65 at Ser^311^ promotes complex formation with CBP, increasing complex binding to the IL-6 promoter [139]. In addition, many inflammatory stimuli induce p65 phosphorylation at Ser^529^/Ser^536^, thereby increasing the transcriptional activity of NF-*κ*B [140–142]. 

In response to cytokines, Thr^254^ of p65 is phosphorylated by an unknown kinase. Once phosphorylated, p65 forms a complex with Pin1, preventing binding to I*κ*B and causing nuclear localization, resulting in greater NF-*κ*B stability and activity [143]. 

The stability of p65 is also regulated by the ubiquitin-proteasome pathway. Treatment of cells with MG132 (a proteasome inhibitor) and His-Ubiquitin resulted in p65 polyubiquitination via interaction with suppressor of cytokine signaling (SOCS)-1. This ubiquitination event was negatively regulated by Pin-1 and increased the stability of p65- and NF-*κ*B-dependent gene expression [137, 143]. 

TNF-*α* was recently reported to induce polyubiquitination of Lys^195^ in p65 and decrease the transcriptional activity of NF-*κ*B by promoting its degradation. This effect of TNF-*α* on p65 appears contradictory but presumably reflects an important regulatory mechanism; that is, persistent activation of p65 by phosphorylation may be terminated by ubiquitination [144].

The expression of glycosyl transferase and NF-*κ*B target genes is regulated by either TNF-*α* or hyperglycemia [145–147]. O-GlcNAcylation of p65, which occurs on Thr^352^, decreases p65 interaction with I*κ*B*α*, resulting in increased NF-*κ*B transcriptional activity during hyperglycemia [146, 147].

### 3.4. Free Fatty Acids-Induced Insulin Resistance in Muscle

Skeletal muscle is one of the main target tissues which respond to insulin and other hormones [148]. Glucose uptake by muscle is stimulated by insulin. In patients with NAFLD, elevated plasma free fatty acids (FFAs) levels are responsible for insulin resistance [149, 150] causing a decrease in the insulin-stimulated glucose uptake, glycogen synthesis [151], and PI3K activity in skeletal muscle [152]. 

Elevated FFA in the blood causes accumulation of triacylglycerol (TG) in the muscle [153], which is shown to be associated with increased intracellular diacylglycerol (DAG), ceramides, and long-chain acyl-coenzyme A (LCA-CoA). These molecules induce insulin resistance by activating serine protein kinase C (PKC) [154]. This kinase inhibits PI3K activities by phosphorylating Ser/Thr residue of IRS-1 causing an inhibition of the insulin-stimulated translocation of the glucose transporter type 4 isoform (GLUT4) [155]. Phosphorylation of I*κ*B by PKC dissociates I*κ*B from NF-*κ*B and thereby translocates NF-*κ*B to nucleus to upregulate proinflammatory TNF*α* gene [154]. NF-*κ*B is linked to fatty acid-induced impairment of insulin action in muscle [156, 157]. 

The increased TG in muscle may be potentially toxic to skeletal muscle presumably because of ROS overproduction which inhibits the insulin-stimulated Akt phosphorylation on Ser residue [158]. ROS also stimulates Thr phosphorylation of JNK, a kinase linked to insulin resistance [159]. An elevated TG is associated with reduced mitochondrial oxidative capacity in skeletal muscles as indicated by lower mitochondrial density, reduced capacity of electron transport, and reduced activities of oxidative enzymes [160]. Further researches are necessary to understand the contribution of PTM of transcription factor in the development of insulin resistance in muscle.

### 3.5. Adipokine Gene Expression and Secretion from Adipose Tissue

Contribution of adipose tissue in the maintenance of whole body insulin sensitivity is critical. Adipogenesis is a tightly regulated process that involves the complicated interrelationship of various transcription factors. One of the pivotal transcription factors is PPAR*γ*, an essential factor of development and function [161, 162]. Hormonal stimuli to the preadipocyte trigger the expression of C/EBP*β* [163] which activates the expression of two master transcription factors, C/EBP*α* and PPAR*γ* [164]. PPAR*γ* can induce adipogenesis in C/EBP*α* ^–/–^ MEFs (mouse embryonic fibroblast) [165], whereas C/EBP*α* is unable to do the same action in PPAR*γ* ^–/–^ MEFs [166]. These results indicate that PPAR*γ* plays a central role in adipogenesis. 

Mitogen-activated protein (MAP) kinase induces the phosphorylation of Ser^112^ of PPAR*γ* resulting in the reduction of transcriptional activity. This observation is supported by a study [167] which showed that PPAR*γ* activity was not decreased by MAP kinase when Ser^112^ was replaced by Ala. Furthermore, treatment of PD98059, an inhibitor of MAP kinase, abolished the phosphorylation of PPAR*γ* [167].

Adipocytes store triglycerides, which are an abundant source of energy, and secrete adipokines such as adiponectin, leptin, resistin, and retinol-binding protein 4 [168]. The expression and secretion of these adipokines are regulated by PTM of various transcription factors in the context of obesity.

One such factor is FOXO1, which regulates adiponectin expression. In FOXO1 haplodeficient animals, adiponectin gene expression is significantly reduced [169]. In fact, two FOXO1 response elements have been identified in the adiponectin promoter [170]. Moreover, SIRT1 was demonstrated to increase the interaction between FOXO1 and C/EBP*α* and enhance subsequent binding to the adiponectin promoter [170]. These results suggest that FOXO1 deacetylation plays an important role in upregulating adiponectin expression. Adiponectin increases insulin sensitivity by promoting fatty acid oxidation in an AMPK and peroxisome proliferator-activated receptor-*α*-dependent manner [171].

The activity of Sp1, a ubiquitously expressed transcription factor that regulates most housekeeping genes, has been shown to be controlled by PTM [172]. In fact, Sp1 was the first transcription factor shown to be O-GlcNAcylated [173]. When O-GlcNAcylated, Sp1 is less phosphorylated and is protected from proteasomal degradation [174]. Presumably, the transcriptional activity of Sp1 may vary depending on the site of O-GlcNAcylation [21].

In 3T3-L1 and primary cultured adipocytes, glucose increases Sp1 *O*-GlcNAcylation and upregulates expression of leptin [175, 176]. Although leptin controls appetite, it is considered a proinflammatory adipokine [177].

Resistin gene expression is increased by glucosamine infusion in rats [178], whereas treatment of 3T3-L1 adipocytes with troglitazone results in decreased gene expression due to a reduction in Sp1 *O*-GlcNAcylation [179]. These experiments indicate that insulin resistance induced by chronic hyperglycemia can be modulated by *O*-GlcNAcylation of Sp1. Interestingly, *O*-GlcNAcylated Sp1 increases the expression of both leptin and resistin [180]. 

## Figures and Tables

**Table 1:** The target genes of the transcription factors.

Transcription factor	Target gene		Reference
	Gene symbol	Description	
FOXO1	*G6PC*	Glucose-6-phosphatase	[36]
	*Pck1*	Phosphoenolpyruvates carboxykinase1	[181]
	*Ppargc1a*	Peroxisome proliferator-activated receptor-coactivator-1 alpha	[38]
	*Pdx1*	Pancreatic and duodenal homeobox 1	[101]
	*NeuroD*	Neurogenic differentiation	[107]
	*MafA*	V-maf (maf musculoaponeurotic fibrosarcoma) oncogene homolog A	[107]
	*ADIPOQ*	Adiponectin	[170]
CREB	*G6pc*	Glucose-6-phosphatease	[57]
	*Pck1*	Phosphoenolpyruvates carboxykinase	[57]
	*Ppargc1a*	Peroxisome proliferator-activated receptor-coactivator-1 alpha	[57]
SREBP-1c	*ACLY*	ATP-citrate lyase	[182, 183]
	*Acaca*	Acetyl-CoA carboxylase alpha	[184]
	*ACACB*	Acetyl-CoA carboxylase beta	[185]
	*Fasn*	Fatty acid synthase	[186]
	*Scd1*	Stearoyl-coenzyme A desaturase 1	[187]
	*Elovl6*	ELOVL fatty acid elongase 6	[188]
ChREBP	*Pklr*	Pyruvate kinase, liver, and RBC	[189]
	*Acc1*	Acetyl-CoA carboxylase 1	[190]
	*Fasn*	Fatty acid synthase	[191]
NF-*κ*B	*TNF-*α**	Tumor necrosis factor alpha	[192]
	*IL-6*	Interleukin 6	[193]
	*MCP-1*	Monocyte chemotactic protein 1	[194]
			
Sp1	*LEP*	Leptin	[195]
	*LETN*	Resistin	[196]
